# A Few Words in Regard to the Sterilization of Instruments

**Published:** 1891-11

**Authors:** Harold C. Ernst


### International Dental Journal.

Vol. XII. November, 1891. No. 11.

### Original Communications.[1]

### A FEW WORDS IN REGARD TO THE STERILIZATION OF INSTRUMENTS.[2]

BY HAROLD C. ERNST, M.D.[3]

Mr. President and Gentlemen,—I have had a great many serious qualms when I have thought about what I should say here to-night, and it has distinctly entered my mind that certainly most of what I shall have to say is not new to any of you, and most probably it is none of it necessary.

The question of the sterilization of instruments is an important one from the modern views of the etiology of disease,—quite as much for the dental surgeon as for the surgeon operating on other parts of the body. The etiology of the different forms of disease resulting from what we call wound-infections has been so cleared up, and we know so much more about them now than we did ten years ago, that the principles upon which sterilization should be based are principles of actual fact and knowledge, and not of theory, and it therefore seemed to me that it might be of interest

---

[1] The editor and publishers are not responsible for the views of authors of papers published in this department, nor for any claim to novelty, or otherwise, that may be made by them. No papers will be received for this department that have appeared in any other journal published in the country.

[2] Before Harvard Odontological Society, 1891.

[3] Instructor in Bacteriology, Harvard University.

to you if I explained briefly, and as plainly as I could, what those principles are.

In considering the sterilization of instruments, the first question which comes up for our investigation is to know exactly what sterilization is; and that can be much more distinctly brought to your minds by the consideration and differentiation of a number of terms that are very apt to be confounded and used interchangeably. We often hear of *germicides*, *antiseptics*, and of compounds, which by virtue of their elements, it is claimed, can be employed for sterilization, that are neither of these, but are simply *deodorants*. These are the three things that are used, and said to be effective, in accomplishing the sterilization of instruments or dressings of infectious wounds. The meaning of the word "sterilization" is the destruction of all forms of microscopic life which may exist upon the instruments or dressings which we wish to use. Now, a deodorant is a very different thing, as you can easily see when the name is brought up by itself; it indicates that it takes away odors, but it does not destroy vitality. The word "antiseptic" is also quite generally supposed to mean germicide; but the action of an antiseptic is merely to prevent the actual carrying on of the processes of germ life by rendering their habitation unfavorable, and yet is not destructive to the vital forces themselves. A *germicide*, on the other hand, is the one thing that we are striving for to obtain perfect sterilization, and it means, of course, something that is actually destructive to germ life of all kinds,—not simply bacteria, but the other forms of organic life that are known to be productive of pathological change. The distinction between these words is not carefully observed, even by those who we would suppose should thoroughly understand it, and we often see in the advertising columns of reputable medical journals something lauded to the skies as a germicide or sterilizing material that is purely and simply a deodorant, and has absolutely no effect in the destruction of the vital forces that one desires to get rid of. It is because of this confusion—and it is through the influence of the commercial manufacturer that the confusion is fostered—that I have laid so much stress upon the differentiation of these terms. This evening, then, we shall have nothing to do with the deodorant, nor with the antiseptic; but we do want to know what is the best germicide for the sterilization of our instruments, and what is the best method of applying that germicide.

Understanding now that by sterilization is meant the actual destruction of any or all forms of vitality that may be in existence upon the instruments or dressings one desires to use, we will find that this sterilization may be completed in a number of different ways. In the first place, it may be accomplished by the use of chemicals, which is a very common and very easy method for certain purposes. In the next place, it may be done by heat; and, as you are aware, the heat that may be employed for sterilizing is of two kinds, the dry and the moist.

In considering the question of the employment of chemicals, we find that we are obliged to use something that is not destructive to the delicate instruments that you use in your every-day work, and, as a rule, chemicals, of which corrosive sublimate is the most efficient, when they are used in sufficient strength to accomplish the destruction of bacteria, not only destroy those bacteria, but also destroy the instrument; so that in accomplishing complete sterilization, chemicals, so far as any that I know of, are out of the question for your purposes.

We come then to the consideration of the employment of heat. Dry heat is extremely effective if it can be used; but the vital objection against its employment for the sterilization of instruments in your profession is, that when used at the actual temperature that is required to effect complete sterilization, it will inevitably take out the temper of any instrument that is subjected to it, which, of course, excludes dry heat as a means for sterilizing as far as your necessities are concerned.

Now, we have excluded chemicals, and also dry heat, and the next question is the employment of moist heat. Of course, you understand that there are certain things that we may require to sterilize that will stand moist heat, and certain other things that will stand dry heat better than moist; but if the moist heat can be used, it is the quickest and most effective means for accomplishing sterilization of which we have any knowledge. Moist heat may be applied in two ways: by boiling, that is, by placing the instrument in a water-bath heated to the boiling-point, or by the use of what is called the steam sterilizer, by the use of which the moist steam, passing over the instruments, accomplishes the sterilization; this latter method is quicker and more effectual than the ordinary boiling, and is also applicable to a wide range of instruments. The common apparatus for applying moist heat, when of steam and not of boiling water, consists simply in a receptacle which holds the water, with a grating over it, upon which the instruments are placed, and a vessel running up and around the instruments, forming a retaining-wall for the steam, having a loosely-fitting cover to put the steam under slight pressure. The very best apparatus for the application of steam heat that I know of is the Arnold Steam Sterilizer. I know it is not usual to mention the name of any particular manufacturer at a meeting of this kind, but this apparatus has decided advantages over any others, and it cannot be spoken of without using the name; its perfections have been recognized not only here but in Europe, where it is now being copied, the manufacturers having stolen the model without giving credit to the inventor. It consists, roughly speaking, of a reservoir that comes immediately over the flame, into which is put only a thin layer of water. When the water has been turned into steam, it passes up into the chamber and over the instruments, then through the holes in the loosely-fitting cover, where it is retained by the outer hood or jacket, and, becoming condensed, drips down the sides again into the reservoir next to the flame, where it is again converted into steam. It does not allow any steam to escape into the room, and so could be placed wherever most convenient. I have had one of them in my laboratory heated to nearly 110° C. without anybody realizing that there was any steam on at all, and by its use the destruction of the most virulent organisms that you could place upon your instruments can be thoroughly completed in about twenty minutes after the steam has begun to pass. The objections that have been raised against steam sterilizing are, that it rusts the instrument, and that there is a possibility of injuring the temper of some of the more delicate. As far as the injury to temper is concerned, I have not seen it occur in my experience, and I believe there is no ground for such objection. With regard to the injury to the polish or rusting of the instruments, unless they are taken out and allowed to cool before drying, there will be little trouble of this kind. It is the quick cooling, and not the application of the steam, that injures the polish of the instrument, and if they are dried quickly after being removed, little danger need be feared from that source. I have thought over the thing a good deal, and considered carefully what I should recommend to you to-night, and I concluded that it would be impossible for me to tell you of any better method for sterilizing your instruments than to use one of the small sized Arnold Steam Sterilizers set over a Bunsen burner, in which your instruments can be placed, either in glass jars or in wire baskets. The glass jars seem to me to be more desirable, and for this purpose the ordinary marmalade jars that we get from the grocer could be utilized, placing a little cotton wool in the necks after you had put in your instruments. After heating, the instruments could be easily removed and thoroughly wiped, and the sterilization be completed quicker and with less possibility of injury to the instruments than by any other method I know of.

Now, so much for the methods of sterilization, and it is all very well to say that such and such things should be done; but I venture to say there would be very little heed paid to it unless it was demonstrated why it should be done.

Of the greatest possible interest in the last few years to those who have followed the course of scientific investigation are the discoveries that have been made, and the absolute proof that has been brought forward that a large number of pathological conditions are due to the presence and rapid growth of the various micro-organisms, most of which are included under the term "bacteria." One of the processes that for a long time was unknown, and was supposed to give ground for the theory of spontaneous generation, was the process of spore formation,—that is, the process of certain bacteria changing from the full-grown rod form to the very thick-membraned, long-resisting spore. The spore is a thing that is specially difficult to get rid of. Bacteria require a certain amount of moisture for a favorable growth; if they are deprived of that moisture, they may produce this resisting stage called spores. In this stage they will resist drying for an indefinite length of time; will resist a considerable amount of heat, and are not easily affected by chemical action. Experiments have been made by Dr. Stone in my laboratory with the bacillus of tuberculosis, and the result shows that the bacilli that are present in every case of pulmonary disease when allowed to dry in the expectoration, on being moistened will resume their activity. In one case the sputum was allowed to dry for three and one-half years, and became so hard that it would take a hammer to break it up; but on being placed in water the organisms were easily detected by the microscope, and their vitality was shown by inoculation experiments. Therefore, drying is not a safeguard against certain diseases, particularly against spore-bearing bacteria. These spores form a middle stage of the life of bacteria, and in that shape the special variety of bacteria to which they belong cannot be differentiated. It cannot be positively stated what special disease they will produce, but their general characteristics are well known, and this fact of tenacious vitality is the most prominent,—the resisting all forms of drying, most chemical reagents, and any moderate action of dry or moist heat.

Now, these facts being settled, to come a little more distinctly to the point that bears upon your branch of the profession, let us see how many of the diseases that are due to infectious agencies—to the bacteria and micro-organisms—may be transferred through the mucous membrane or the saliva, and how much we have to fear from them.

The most widespread disease that we have to contend with, and that is more destructive to human life than any other, is tuberculosis. It is now very generally conceded that tuberculosis is due to a micro-organism. This micro-organism is unquestionably possessed of the most resisting powers,—possibly the anthrax bacillus surpasses it somewhat, but not much,—and tuberculosis has its origin quite as frequently through the mouth as it does in other parts, and very frequently the cause of the disease is found in the saliva of the mouth, especially in pulmonary tuberculosis. Of course, it is impossible to detect the bacilli without the aid of the microscope; nevertheless the infectious agent is probably present in a large number of your patients, pulmonary tuberculosis being so prevalent. I know that it is present in all cases with which I am familiar, and it is fair to presume that a great many of my patients are also your patients. Therefore, tuberculosis is the first thing for you to be on your guard against, and it is against the possibility of infection that your patients are to be protected.

In the second place, not so frequent, comes the disease that is unfortunately too prevalent among our patients, and that is syphilis. It is one of the most dreadful diseases with which humanity can be affected, and its symptoms, as they occur upon the mucous membrane of the mouth and the inside of the cheeks, are altogether too common and well known by you to require from me any description. It is not so generally acknowledged that this is a germ disease, nevertheless enough of its characteristics have been learned by experiment and observation for us to be perfectly sure that it requires the same precautions against the possibility of infection as does tuberculosis. Here, then, are two diseases that may be transferred upon instruments used about the mouth.

A third, and one that by my belief is not as commonly recognized as it should be, even by members of the medical profession, is a disease that is known in the West as "break-jaw." It is due to a fungus, not a bacterium. It has been given the name of "actinomycosis," and the symptom that makes it known in cattle is the fact that it starts as an enlargement and suppuration of the submaxillary glands, and in all cases that have occurred in man it has also made its appearance in the glands under the inferior maxilla. So far as it has been possible to learn of this disease, it has always gained access through the mucous membrane rather than through the skin, and it is my belief that there are many cases of that disease occurring in man that are generally classified as scrofulous disease, especially among children. Then, certainly, this is a disease against which these principles of sterilization should be directed.

Another disease, an experimental disease, because it has been studied principally by experiment, is due to the micrococcus Pasteuri (M. of Sputum Septicæmia). It produces a tremendous suppuration upon subcutaneous injection, and it is probably the cause of the bad results from bites and that class of wounds, and that give the surgeon so much trouble. The organism is destroyed with a great deal of difficulty by drying, and occurs in the mouths of apparently healthy persons.

Another disease, which does not occur commonly among your patients, from the fact that when a person is afflicted with it he is generally in bed, and yet that in its early stages might very readily be transferred, is diphtheria. It is now known that the sputum of a person ill with diphtheria is full of micro-organisms called the diphtheria-bacillus. This is also one of the rod-shaped bacteria that must be looked out for.

In order not to make this catalogue endless,—one of your profession, Dr. Miller, of Berlin, has completed it,—I will simply say that the remaining active micro-organisms that may be found in the mouth are the ordinary bacteria of suppuration, and two or three others; and as these are not infrequently found in persons who are apparently healthy, it is of course impossible to say on mere inspection that these bacteria are not present in full activity. Of course, the inference is that they must be destroyed before the instruments are used a second time.

Now, of the bacteria that I have named, with the exception of tuberculosis, syphilis, and actinomycosis, ordinarily careful washing and drying might do. It would be pretty certain to be sufficient for the less resistant kinds of bacteria; but for these three it seems to me that the possible resistance of any one of them furnishes a perfectly justifiable basis for a word of warning in regard to the sterilization of instruments. In all that I have said about the necessity for this caution, I hope you will not feel that I am trying to make an unnecessary scare. I have brought a few lantern slides to give you some idea of what these organisms look like.

(Lantern slides shown.)

